# Vaccine and Drug Ontology Studies (VDOS 2014)

**DOI:** 10.1186/s13326-015-0039-8

**Published:** 2016-02-25

**Authors:** Cui Tao, Yongqun He, Sivaram Arabandi

**Affiliations:** University of Texas, Health Science Center at Houston, of Biomedical Informatics, Houston, TX USA; Unit for Laboratory Animal Medicine, Department of Microbiology and Immunology, and Center for Computational Medicine and Bioinformatics, University of Michigan Medical School, Ann Arbor, MI USA; Ontopro LLC, Houston, TX USA

## Abstract

The “Vaccine and Drug Ontology Studies” (VDOS) international workshop series focuses on vaccine- and drug-related ontology modeling and applications. Drugs and vaccines have been critical to prevent and treat human and animal diseases. Work in both (drugs and vaccines) areas is closely related - from preclinical research and development to manufacturing, clinical trials, government approval and regulation, and post-licensure usage surveillance and monitoring. Over the last decade, tremendous efforts have been made in the biomedical ontology community to ontologically represent various areas associated with vaccines and drugs – extending existing clinical terminology systems such as SNOMED, RxNorm, NDF-RT, and MedDRA, developing new models such as the Vaccine Ontology (VO) and Ontology of Adverse Events (OAE), vernacular medical terminologies such as the Consumer Health Vocabulary (CHV). The VDOS workshop series provides a platform for discussing innovative solutions as well as the challenges in the development and applications of biomedical ontologies for representing and analyzing drugs and vaccines, their administration, host immune responses, adverse events, and other related topics. The five full-length papers included in this 2014 thematic issue focus on two main themes: (i) General vaccine/drug-related ontology development and exploration, and (ii) Interaction and network-related ontology studies.

## Introduction and background

Drugs and vaccines have been critical to prevent and treat human and animal diseases. Work in both (drugs and vaccines) areas is closely related - from preclinical research and development to manufacturing, clinical trials, government approval and regulation, and post-licensure usage surveillance and monitoring. Ontologies can serve important roles in managing, normalizing, sharing, and leveraging drug and vaccine relevant data. The 2014 “Vaccine and Drug Ontology Studies” workshop (VDOS 2014) was an international forum for researchers to identify, propose, and discuss solutions for important research problems in ontology representation and analysis of vaccine and drug development, administration, function mechanisms, safety, and education.

VDOS 2014 was held on October 7, 2014, in Houston, Texas. This workshop was part of the Fifth International Conference on Biomedical Ontology (ICBO 2014). The workshop attracted interests from many international attendees, including paper presenters, academic and government scientists, postdoctoral fellows, and graduate students. After a rigorous peer review process (all submissions have been reviewed by at least three independent reviewers), five full-length papers were accepted for proceeding paper publications and oral presentations in the workshop. After additional reviewing by the independent reviewers, workshop co-organizers, and the journal editors, the selected five full-length papers were extended and accepted for publication in the thematic track of the Journal of Biomedical Semantics (JBMS).

The VDOS-2014 workshop is the 3^rd^ in this series. The first workshop of the series was organized as the “Vaccine and Drug Ontology in the Study of Mechanism and Effect” workshop (VDOSME 2012) [1] on July 21, 2012, in Graz, Austria, as part of the third International Conference on Biomedical Ontology (ICBO 2012). In 2013, the name has been changed to “Vaccine and Drug Ontology Studies (VDOS)” to reflect the expansion in the scope to more than just mechanism and effect. The workshop series also covers vaccine and drug-related clinical data representation and analysis, including clinically reported vaccine and drug adverse events. VDOS 2013 was held on July 7, 2013, in Montreal, Qc, Canada [2].

## Summary of selected papers in the thematic issue

The five papers selected for this thematic issue are extended versions of the original full-length papers presented at the VDOS 2014. This year the workshop focuses on two main themes: (i) General vaccine/drug-related ontology development and exploration, and (ii) Interaction and network-related ontology studies.

In the area of general vaccine/drug-related ontology development and exploration, Winnenburg and Bodenreider introduced their approach on identifying common class-level signals for every individual drug in a class [3]. They created a dataset of Adverse Drug Events (ADE) based on information extracted from PubMed abstracts and aggregated drugs into Anatomical Therapeutic Chemical (ATC) classes and ADEs into Medical Subject Headings (MeSH) terms. They then applied a visual approach to uncover known associations and a computational approach to systematically analyze the associations between drug classes and ADEs. Amith and Tao introduced their Vaccine Information Statement Ontology (VISO), which is a comprehensive vaccine information ontology that can support personal health information applications using patient-consumer lexicon, and lead to outcomes that can improve patient education [4]. The current VISO contains the knowledge included in the Vaccine Information Statement (VIS) documents collected from the USA Centers for Disease Control and Prevention (CDC) website. The overarching goal of the VISO is to positively influence the development of intelligent ontology-driven applications and mitigate the knowledge gap that often exists in patients seeking accurate and reliable information but encountering complex or inaccurate sources.

In the area of interaction and network-related ontology studies, Peters et al. investigated the drug –drug interaction (DDI) information in two publically available drug resources, NDF-RT and DrugBank [5]. Their study indicated that the overlaps of DDIs between the two resources are limited. Further studies need to be conducted to either determine how to best leverage the DDI information included in these two resources in clinical and translational applications. Hur et al. introduced their work on the development of the interaction Network Ontology (INO) with an aim to classify over 800 interaction keywords for literature mining various interactions between genes and proteins [6]. Based on the INO-classified interaction type hierarchy and the Vaccine Ontology (VO)-based vaccine hierarchy, a modified Fisher’s exact test on PubMed literature mined results was established to successfully analyze significantly over- and under-represented enriched gene-gene interaction types within the vaccine domain. This study demonstrated that hierarchical ontological definitions facilitate novel approaches for literature mining and analysis of gene interaction networks. Zhang et al. introduce a novel approach that combines ontologies and network analysis technologies for studying sex-associated patterns in vaccine adverse events [7]. The authors leverage data downloaded from the Vaccine Adverse Event Report System (VAERS) and constructed a condition-specific association network. The outcome of this research can potentially provide guidance on sex-specific dose recommendations in personalized vaccinology.

## Workshop presentations and discussions

In the 2014 VDOS workshop, the five full-length papers described above were orally presented. In addition, the program also included a keynote speech and a podium abstract presentation. The keynote speech was given by Dr. Khalid F. Almoosa, Associate Professor of Medicine at the Department of Internal Medicine, UTHealth Medical School, Houston, TX. Dr. Almoosa also serves as the Vice-Chair for Healthcare Quality and the Director of Interprofessional Collaboration at UTHealth. In his keynote, Dr. Almoosa discussed important issues in healthcare quality and safety and the roles of vaccine and drug safety in healthcare quality management. For the podium abstract presentation, Dr. Yongqun He provided updates on the development of the Ontology of Adverse Events (OAE) and its applications. An in-depth discussion of the OAE is available in the recently published article in Journal of Biomedical Semantics [8].

The discussion session of the workshop focused on the topic of “Drug and vaccine ontology informatics”, we first discussed the similarities and differences between the adverse event ontologies in the Open Biological and Biomedical Ontologies (OBO) domain (e.g., OAE and AERO) and non-OBO style ontologies (e.g., MedDRA and SNOMED). The necessity and possible approaches on how to bridge OBO and non-OBO ontologies were explored. Furthermore, the participants of the workshop discussed ontology-based clinical and experimental data processing and analysis, including ontology-supported literature mining of biomedical and electronic health records. We all agreed to continue the workshop in 2015 which would be held in conjunction with the ICBO-2015 conference at Lisbon, Portugal, in the end of July 2015.

Overall, the VDOS 2014 workshop was well received with positive feedbacks. The VDOS workshop series have served and will continue to serve a platform for biomedical and clinical researchers to discuss ontology-relevant issues and progress in drug and vaccine research and applications.
